# Facile Construction of Superhydrophobic Surfaces by Coating Fluoroalkylsilane/Silica Composite on a Modified Hierarchical Structure of Wood

**DOI:** 10.3390/polym12040813

**Published:** 2020-04-04

**Authors:** Jiajie Wang, Yingzhuo Lu, Qindan Chu, Chaoliang Ma, Lianrun Cai, Zhehong Shen, Hao Chen

**Affiliations:** School of Engineering, Zhejiang A&F University, Hangzhou 311300, China

**Keywords:** superhydrophobic surfaces, self-healing, natural hierarchical microstructures, wood

## Abstract

Constructing superhydrophobic surfaces by simple and low-cost methods remains a challenge in achieving the large-scale commercial application of superhydrophobic materials. Herein, a facile two-step process is presented to produce a self-healing superhydrophobic surface on wood to improve water and mildew resistance. In this process, the natural hierarchical structure of wood is firstly modified by sanding with sandpaper to obtain an appropriate micro/nano composite structure on the surface, then a fluoroalkylsilane/silica composite suspension is cast and dried on the wood surface to produce the superhydrophobic surface. Due to the full use of the natural hierarchical structure of wood, the whole process does not need complicated equipment or complex procedures to construct the micro/nano composite structure. Moreover, only a very low content of inorganic matter is needed to achieve superhydrophobicity. Encouragingly, the as-obtained superhydrophobic surface exhibits good resistance to abrasion. The superhydrophobicity can still be maintained after 45 abrasion cycles under the pressure of 3.5 KPa and this surface can spontaneously recover its superhydrophobicity at room temperature by self-healing upon damage. Moreover, its self-healing ability can be restored by spraying or casting the fluoroalkylsilane/silica composite suspension onto this surface to replenish the depleted healing agents. When used for wood protection, this superhydrophobic surface greatly improves the water and mildew resistance of wood, thereby prolonging the service life of wood-based materials.

## 1. Introduction

The fabrication of superhydrophobic surfaces with water contact angles (CAs) larger than 150° and sliding angles less than 10° has attracted extensive research attention worldwide due to its great potential in both theoretical research and practical applications, such as self-cleaning [1], anti-fouling [2], durable antibacterial uses [3], oil/water separation [4,5], gas sensing and droplet manipulation [6]. The superhydrophobic property of the surface is controlled by its chemical composition and topography. The cooperation of micro/nano scale hierarchical structures with low-surface energy materials has been the main strategy to fabricate superhydrophobic surfaces [7]. Through years of extensive efforts, many chemical and physical methods that generate superhydrophobic surfaces have been developed, such as plasma polymerization/etching [8], chemical vapor deposition [9,10,11,12], solvent-mediated phase separation [13], and polymer self-assembly [14,15,16]. However, some superhydrophobic surfaces are apt to lose their superhydrophobicity during practical applications since the artificial micro/nano hierarchical architectures are susceptible to being damaged after a slight scratch, abrasion, or even brief contact with fingers, which has hampered their widespread application. Recently, although many researchers have reported the successful fabrication of mechanically durable superhydrophobic surfaces [17,18,19,20,21,22,23], it is still highly desired to construct superhydrophobic surfaces by simple and low-cost methods to achieve the large-scale commercial application of superhydrophobic materials.

Wood, as a renewable resource, has great potential in decorative fields to substitute steel, stone, glass, minerals, and synthetic resin. Nevertheless, because it contains many hydrophilic groups such as hydroxyl groups, the wood is apt to absorb water, which can result in dimensional instability and attacks by microorganisms including decay fungi and mildew [24,25,26]. Therefore, it is of great practical significance to construct a high-performance superhydrophobic surface for the wood.

In this work, we have described a facile process to fabricate a robust self-healing superhydrophobic surface on wood with improved water and mildew resistance. In this process, the natural hierarchical structure of wood was firstly modified by sanding with sandpaper to obtain an appropriate micro/nano composite structure on the surface, then a fluoroalkylsilane/silica composite suspension was cast and dried on the wood surface to produce the superhydrophobic surface. Due to the full use of the natural hierarchical structure of wood, the whole process does not need complicated equipment or complex procedures to construct the micro/nano composite structure. Moreover, only a very low content of inorganic matter is needed to achieve superhydrophobicity. Therefore, the as-obtained superhydrophobic surface is transparent and unveils the natural grains and textures of the original surface. More importantly, benefiting from the unique micro/nano composite structure originated from the natural hierarchical structure of wood, the superhydrophobic surface exhibits robust superhydrophobic performance against physical damages. Furthermore, owing to the intrinsic porous structures of wood, the superhydrophobic surface can preserve a large number of fluoroalkylsilane moieties as the healing agent in the wood pores such as cell cavities and grooves. Once the primary top fluoroalkylsilane layer is decomposed or scratched, the internally-preserved healing agent in the wood pores will migrate to the surface to heal the superhydrophobicity at room temperature. Thus, the superhydrophobic surface exhibits good self-healing ability when damaged. And this self-healing ability can be restored by spraying or casting the fluoroalkylsilane/silica composite suspension onto the wood surface to replenish the depleted healing agents. In addition, when used for wood protection, this superhydrophobic surface greatly improves the water and mildew resistance of wood, thereby prolonging the service life of wood-based materials.

## 2. Materials and Methods

### 2.1. Materials

Ethanol (AR, ≥99.5%), nano fumed silica (99.8wt%, diameter: 7-40nm) and acetic acid (AR, ≥99.5%) were purchased from Shanghai Chemical Reagent Co. (China). Perfluorooctyltriethoxysilane (KH1322) and Chinese fir wood were kindly donated by Zhejiang Longyou Wood Bond Co. (China). Woodblocks with a size of 40 × 35 × 10 mm (longitudinal × radial × tangential) were obtained from the sapwood of Chinese fir (*Cunninghamia lanceolata*). 

### 2.2. Preparation of Fluoroalkylsilane/Silica Suspension

Typically, 0.2 g of KH1322 (as a fluoroalkylsilane) was dissolved in 10 g of ethanol. The solution was mildly stirred until it became homogeneous and transparent. Then, 4 mg silica nanoparticles (the ratio of silica to KH1322 was 2%) were added into the previous solution and ultrasonically treated for one minute until the resulting fluoroalkylsilane/silica composite suspension was homogeneous.

### 2.3. Preparation of Superhydrophobic Surfaces 

The woodblock was first sanded with 240-grit sandpaper under a pressure of 40 KN∙ m^−2^ until a visible clean smooth surface was obtained. In this way, the natural hierarchical structure of wood was modified to obtain an appropriate micro/nano composite structure on the surface. Then, the above suspension was cast onto the wood surface (40 mm × 35 mm) and heated in the oven for a certain period of time to form superhydrophobic surfaces. The dosage of KH1322 plus silica on the wood surface was calculated to be about 4 g∙ m^−2^. Unless specified, the heating temperature was 120 °C, and the duration of heating was 2 hours. 

### 2.4. Characterization

The surface morphology was observed by scanning electronic microscopy (SEM). The VK-X 3D optical laser microscope system (OPM) was used to characterize the three-dimensional surface morphology. The roughness factor (Ra) was calculated according to JIS B0601:1994. The measurement of the surface wettability was performed using a dynamic contact angle (CA) testing instrument (OCA40, Filderstadt, Germany). CA was recorded at 30 seconds after a droplet of liquids (5 μL) was placed on the surface. The sliding angle (SA) was measured by recording the tilt angle of the sample platform at which a liquid droplet (10 μL) started to roll off the surface. Average water CAs were obtained on radial sections by measuring the same sample at six different positions. Fourier transform infrared spectroscopy (FTIR) measurements were carried out using a Nicolet Nexus 470 spectrometer (Thermo Fisher, Waltham, MA, USA). The chemical composition of the wood surface was measured by X-ray photoelectron spectroscopy (XPS, Perkin-Elmer PHI 5000C ECSA, Waltham, MA, USA). The abrasion resistance of the superhydrophobic surface was evaluated by dragging a piece of 240-grit sandpaper under 500 g of weight in one direction with a speed of 1 cm s^−1^ at a distance of 10 cm per cycle. Digital pictures were captured using a Canon Power Shot A 95 digital camera, and the optical images were obtained with the VK-9710 microscope. Unless specified, the water CA tests and characterizations such as SEM, XPS, FTIR, and OPM were carried on the radial sections of wood. 

## 3. Results and Discussion

### 3.1. Fabrication of Superhydrophobic Surfaces

**Scheme 1:**
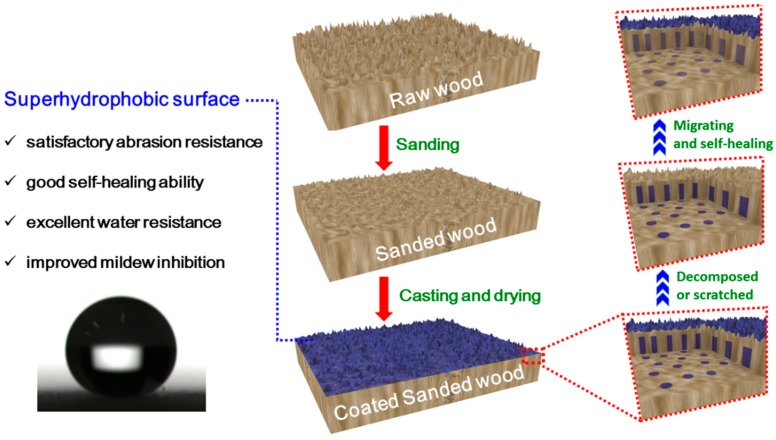
Schematic procedure for the preparation of the superhydrophobic wood surface (middle) and its self-healing mechanism (right).

The process to fabricate the superhydrophobic surface was shown in Scheme 1. The raw wood was first sanded with 240-grit sandpapers to modify the natural hierarchical structure of the wood to obtain an appropriate micro/nano composite structure on the surface. Then, the homogeneous suspension of KH1322 fluoroalkylsilane and silica was cast onto the surface of the sanded wood and heated in an oven or room-dried for a certain period of time to form the superhydrophobic surface. The resulting material is denoted as coated sanded wood. 

**Figure 1:**
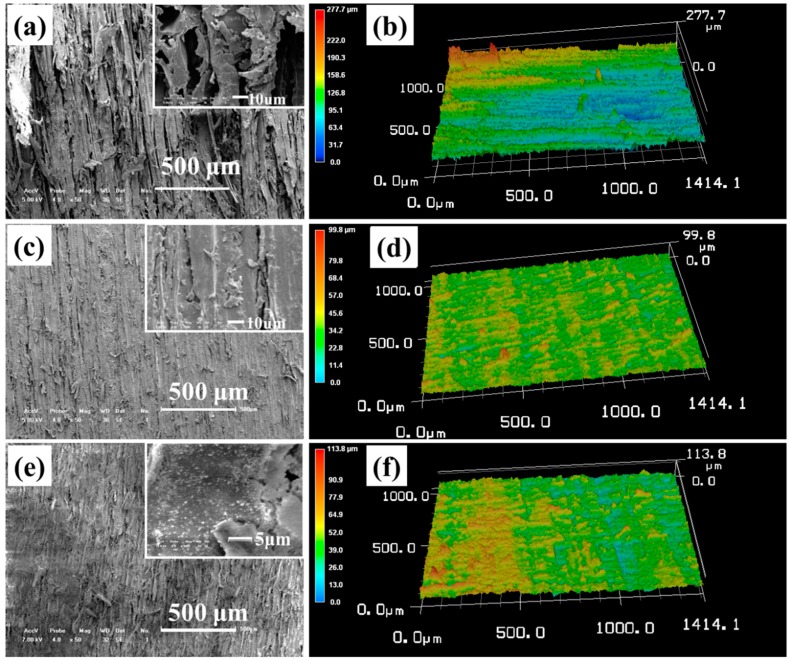
(**a**) Scanning electronic microscopy (SEM) and (**b**) 3D optical laser microscope system (OPM) images of raw wood. (**c**) SEM and (**d**) 3D OPM images of sanded wood. The insets are magnified SEM images with scale bars at 10 μm. (**e**) SEM and (**f**) 3D OPM image of sanded wood coated with KH1322 fluoroalkylsilane and silica.

Figure 1a displays that the natural hierarchical structure of raw wood is highly uneven with a maximum height of more than 200 μm (Figure 1b) and a roughness factor (Ra) of 38 μm. Moreover, some pores were found to be too large (the inset of Figure 1a) to support the weight of the water droplet that resulted in a homogeneous wetting, namely, Wenzel states [27]. Thus, this natural hierarchical structure is not applicable to directly construct the superhydrophobic surface. As expected, when the fluoroalkylsilane/silica suspension was cast and dried on the surface of this raw wood, the water CA on the resultant surface was only 132°, hence the superhydrophobicity cannot be achieved. Figure 1c demonstrates that a relatively uniform micro/nano hierarchical structure can be obtained by sanding the raw wood with 240-grit sandpapers. Figure 1d manifests that the maximum height and Ra of the modified surface structure of sanded wood decreased to be less than 100 μm and 5.69 μm, respectively. The air in the uniform pores can reduce contact areas between water droplets and surface, resulting in the Cassie–Baxter state [28]. Benefiting from this state, the coated surface obtained by drying the fluoroalkylsilane/silica suspension on the sanded wood exhibited a typical superhydrophobic feature with a water CA of 160° and a sliding angle below 10°.

To understand the detailed formation mechanism of this superhydrophobic surface, its three-dimensional surface morphology was investigated by SEM and OPM. The low magnification SEM image of coated sanded wood in Figure 1e displays a similar micromorphology compared with that of the sanded wood without coating (Figure 1c). However, we can find some new particles on the surface in its high magnification SEM image (the inset of Figure 1e). These particles should be the silane oligomers produced by the KH1322 hydrolysis and silica particles. Their emergence led to an increase in the roughness. As a result, both the maximum height (113.8 μm) and Ra (10.21 μm) of coated sanded wood in the OPM image of Figure 1f have a slight increase compared with the data of the sanded wood without coating (Figure 1d). 

**Figure 2:**
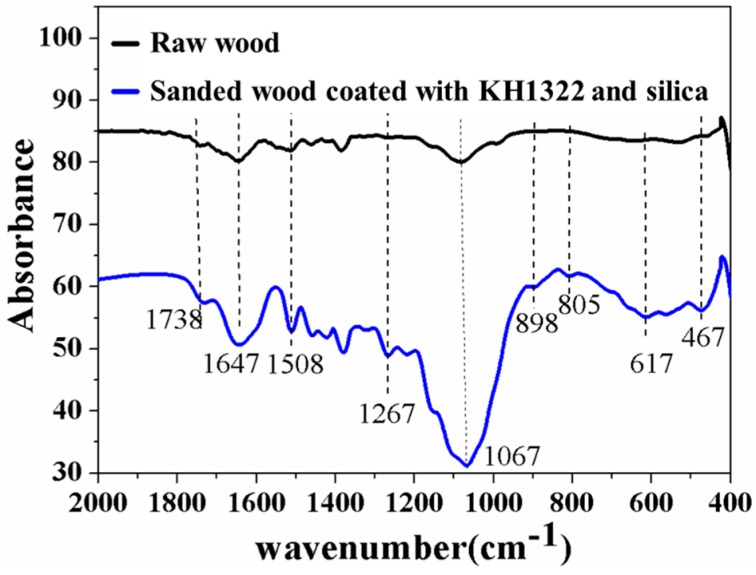
Comparison of Fourier transform infrared spectroscopy (FTIR) spectra between raw wood and sanded wood coated with KH1322 fluoroalkylsilane and silica.

Usually, the raw wood surface is hydrophilic due to its naturally porous structure with abundant hydroxyl groups [25]. Thus, the chemical polarity of the surface must be changed to achieve superhydrophobicity. The comparison of FITR spectra in Figure 2 reveals that there are five new absorbance bands for the coated sanded wood compared with the raw wood. Among them, two peaks at 1267 and 617 cm^−1^ can be attributed to the vibrations of CF_3_ and CF groups [29]. The other three absorbance peaks at 898, 805 and 467 cm^−1^ are ascribed to the vibrations of Si-O. It is believed that the molecular chains with these hydrophobic groups combine with the uniform micro/nano hierarchical structure of the sanded wood to achieve the construction of the superhydrophobic wood surface.

### 3.2. The Effect of Silica on the Surface Wettability 

In this work, the silica was added to prepare the hydrophobic surface. Thus, the effect of silica content on the surface wettability was investigated. It was found that the water CA increased from 152° to 160° when the ratio of silica to KH1322 increased from 0.5% to 2%. However, when the silica ratio further elevated from 2% to 4%, the water CA remained nearly unchanged at 160°. The silica ratio and the total consumption of KH1322 plus silica for superhydrophobic surfaces in the present study were much less than those in previous works [29]. The minimum silica ratio used in this work reached as low as 0.5%, and the total consumption per unit area of KH1322 plus silica was only 4 g∙m^−2^, indicating a low-cost characteristic of the preparation method.

**Figure 3:**
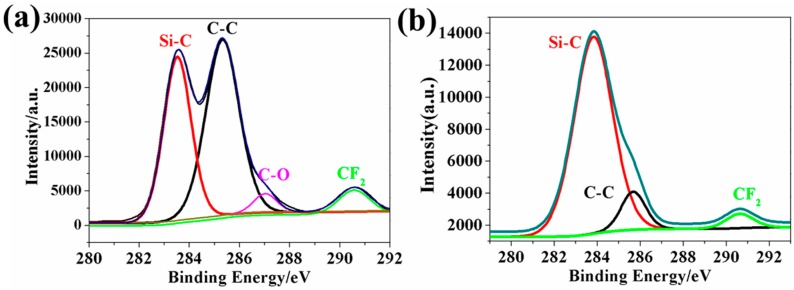
C 1s X-ray photoelectron spectroscopy (XPS) spectra of (**a**) sanded wood coated with KH1322 and 0.5% SiO_2_ and (**b**) sanded wood coated with KH1322 and 2.0% SiO_2_.

Unlike previously reported method based on silica to prepare the superhydrophobic surface, the silica plays a major role in accelerating the hydrolysis and condensation of KH1322 in this work, rather than building micro-nano morphologies. Therefore, the superhydrophobic surface could be obtained even under a low silica ratio of 0.5%. The detailed reason for the silica ratio affecting the surface wettability can be explained as follows. When the silica particles were added into KH1322 solutions, the hydrolysis of KH1322 was developed more quickly. The hydrophilic molecular chains containing C-O groups reduced and the wood surface was covered by more hydrophobic molecular chains containing Si-O groups, resulting in an increase in the water CAs correspondingly. When the silica feeding ratio further increased to 2%, almost all -OC_2_H_5_ groups from KH1322 were hydrolyzed to generate ethanol, which was completely removed from the wood surface during the drying process, thus the C-O groups could hardly be detected. At this point, the number of hydrophobic groups reached the maximum value. In order to confirm the above assumptions, XPS was used to analyze the element change on the wood surfaces. In Figure 3a, the C 1s XPS broad peak of sanded wood coated with KH1322 and 0.5% SiO_2_ can be fitted to four peaks with binding energies of 283.8, 285.6, 287.3 and 290.9 eV, which can be indexed to the functional groups of Si-C, C-C, C-O, and CF_2_, respectively [30]. Here, the ratio of hydrophilic carbons (C-O groups) to all carbon atoms was calculated to be 3.46% based on the peak areas in Figure 3a. However, for the sanded wood coated with KH1322 and 2% silica, the hydrophobic C atoms (Si-C, C-C, and CF_2_ groups) can account for nearly 100% of all C atoms. Hence, the additional increase in the silica ratio to 4% no longer increased the ratio of hydrophobic groups, so the water CAs did not increase further.

### 3.3. The Effect of Drying Temperatures on Surface Wettability

**Figure 4:**
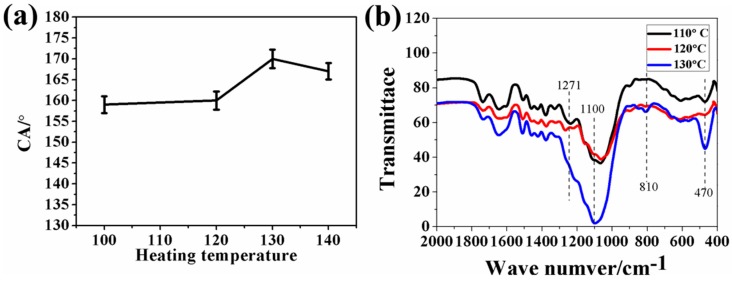
(**a**) The water contact angles (Cas) of the surfaces of coated sanded wood obtained at different drying temperatures, (**b**) Comparison of FTIR spectra of sanded woods coated with KH1322 fluoroalkylsilane and silica obtained at different drying temperatures.

Usually, the drying temperature is a key factor that affects the formation of the coating. Therefore, the influence of drying temperature on the surface wettability was also researched. As shown in Figure 4a, the water CA increased from 157° and 160° to 170° with the elevation of the drying temperature from 100 °C and 120 °C to 130 °C. When the drying temperature was increased again to 140 °C, the water CA decreased slightly from 170° to 167°. The previous CA increase should result from the reduction of hydrophilic C-O groups. It is believed that the increase in the drying temperature from 100 °C to 120 °C accelerated the hydrolysis of KH1322. More -OC_2_H_5_ groups of KH1322 reacted with the water to release ethanol vapour. Therefore, the hydrophilic C-O groups reduced correspondingly, as illustrated by the diminished peak intensity of C-O groups at 1271 cm^−1^ (Figure 4b). When the drying temperature was further elevated to 130 °C, almost all -OC_2_H_5_ groups of KH1322 were hydrolyzed to generate ethanol vapour. The virtual disappearance of the C-O signal in Figure 4b demonstrates the removal of more hydrophilic groups. Moreover, the adsorption of hydrophobic groups (Si-O at 810 and 470 cm^−1^) reached the maximum signal. These facilitate the formation of the superhydrophobic surface with a larger water CA. It could be inferred that the drying temperature of 130 °C is high enough to achieve the complete hydrolysis of KH1322. Thus, when the temperature further increased to 140 °C, the ratio of hydrophobic groups on the wood surfaces no longer increased, with the result that the water CA would not increase correspondingly.

Considering that hours of high-temperature drying may induce damages to some wood, the drying at room temperature (25 °C) was also tried to prepare the hydrophobic surface. It was found that the water CA on the as-obtained surface increased from 75° to 155° when the drying time was extended from 2 hours to 8 hours. This result revealed that the fabrication of a superhydrophobic surface on the wood can also be achieved at room temperature when the drying time is long enough. This suggests that the method can be extended to produce superhydrophobic surfaces for thermally unstable wood products.

### 3.4. Mechanical Robustness and Self-Healing of Superhydrophobic Surfaces

**Figure 5:**
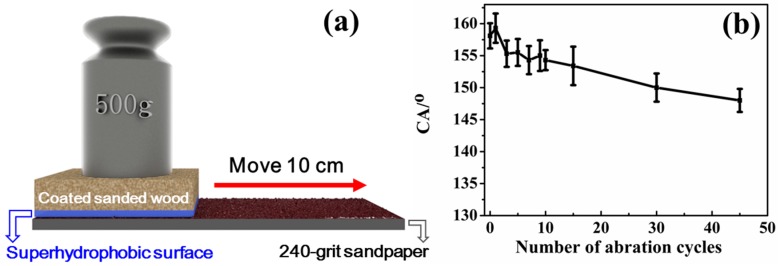
(**a**) Illustration of the abrasion resistance test for the superhydrophobic surface. (**b**) Water CAs as a function of the number of abrasion cycles.

One obstacle to the widespread practical applications of superhydrophobic surfaces is the lack of enough robustness. To investigate the abrasion resistance of the as-obtained superhydrophobic surface, under the loading of 500 grams (pressure: 3.5 KPa), the hydrophobic surface of the coated sanded wood was oriented toward 240-grit sandpaper and moved a distance of 10 cm (each cycle) in a straight line, as shown in Figure 5a. Then, the water CAs of the resulting wood surface were tested. This process was repeated for 45 cycles, and the test results of water CAs were recorded in Figure 5b. One can see that after 45 cycles, the water CA was able to retain a high value of 153°, and the sliding angle was still less than 10°, indicating that the surface of the coated sanded wood maintained its superhydrophobicity. These results demonstrate the satisfactory abrasion resistance of the as-prepared superhydrophobic surface.

**Figure 6:**
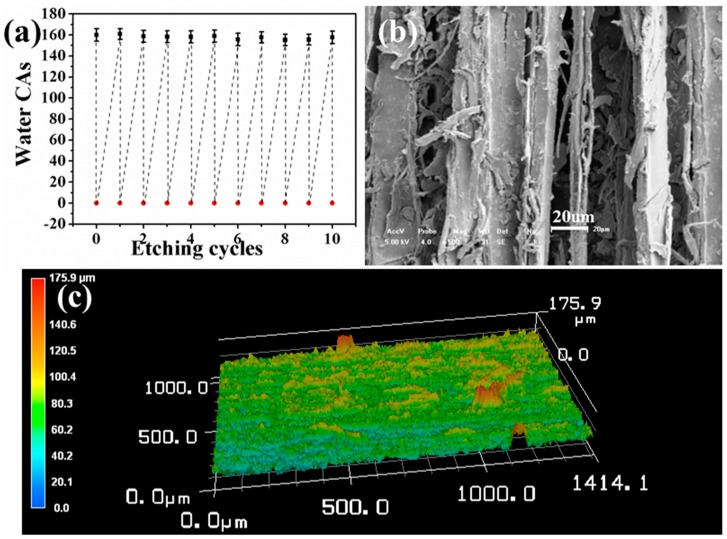
(**a**) Water CAs of the surface after repeated alkaline etching and self-healing at room temperature. (**b**) SEM and (**c**) 3D OPM images of the surface of coated sanded wood after alkaline etching.

As an important indicator affecting service life, the self-healing ability of the superhydrophobic surface was examined by alkali etching. After it was soaked in alkali solution for two hours, the water CA of the resulting surface decreased from 160° to 0°, indicating that this surface had lost its superhydrophobicity. However, after this damaged surface was exposed in an ambient environment for 8 hours, its water CA and sliding angles returned to 160° and <10°, respectively, demonstrating that the original superhydrophobicity of the damaged surface was restored. The recovery of superhydrophobicity should be attributed to the unique inherent porous structure of wood. This intrinsic porous structure can preserve a large number of fluoroalkylsilane moieties as healing agents in wood pores (such as cell cavities and grooves) beneath the superhydrophobic surface. Once the primary top fluoroalkylsilane layer is decomposed or scratched, the internal preserved healing agents in the cell cavities and grooves will migrate to the wood surface (as illustrated by the right part of Scheme 1) to minimize the surface free energy due to the exposure to the hydrophobic air [31,32]. As a result, the superhydrophobicity is healed at room temperature. In this way, the damaged surface recovered its superhydrophobicity at room temperature when the time duration was long enough for the healing agents in pores to migrate onto the wood surface. As displayed in Figure 6a, the etching–healing process can be repeated for nine cycles without decreasing the superhydrophobicity of the self-healed surface. It is believed that the robust internal porous microstructure of wood is essential for the recovery of superhydrophobicity as shown in Figure 6b,c. In addition, it was found that when the temperature increased to 100 °C and 140 °C, the recovery time of the superhydrophobicity reduced to 2 h and 1 h, respectively. This indicates that this self-healing is temperature-dependent, with a more accelerated self-healing process under higher temperatures and vice versa. At present, the self-healing of the as-constructed superhydrophobic surface can be accomplished at room temperature. Thus, this self-healing can be applied to some wooden decorative materials that are sensitive to heat and ultraviolet radiation. Furthermore, if repeated self-healing runs out of healing agents, the suspension of KH1322 fluoroalkylsilane and silica can be cast and dried onto the surface again to replenish the healing agents.

### 3.5. Improvement in Water and Mildew Resistance of Superhydrophobic Surfaces

**Figure 7:**
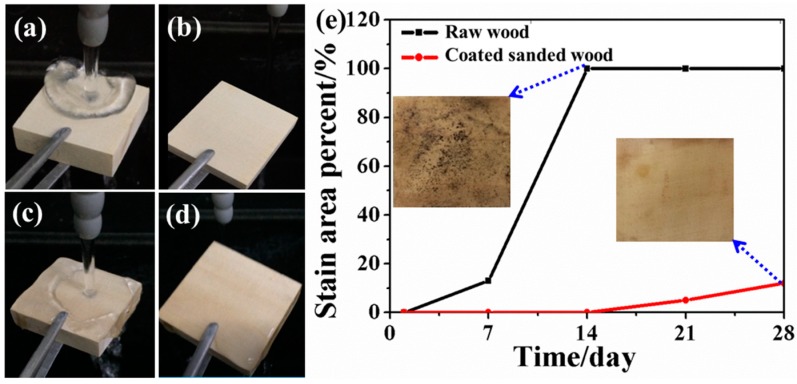
Photographs of the superhydrophobic wood surface (**a**) during water washing and (**b**) after water washing. Photographs of the raw wood surface (**c**) during water washing and (**d**) after water washing. (**e**) Comparison of mildew infection on the surface of raw wood and coated sanded wood in a humid environment.

As a natural biomass material with many hydrophilic groups, raw wood is apt to absorb water and swell in dimension when in contact with water [24]. In order to simulate the rinse effect of rainwater on the surface of outdoor wood products, the as-obtained superhydrophobic surface of coated sanded wood was washed by running water for 20 minutes (Figure 7a). No residual water can be observed on the superhydrophobic surface after this process (Figure 7b). By contrast, excessive water remained on the surface of raw wood after the same treatment (Figure 7c–d). These results indicate the excellent water resistance of the as-constructed superhydrophobic surface.

**Figure 8:**
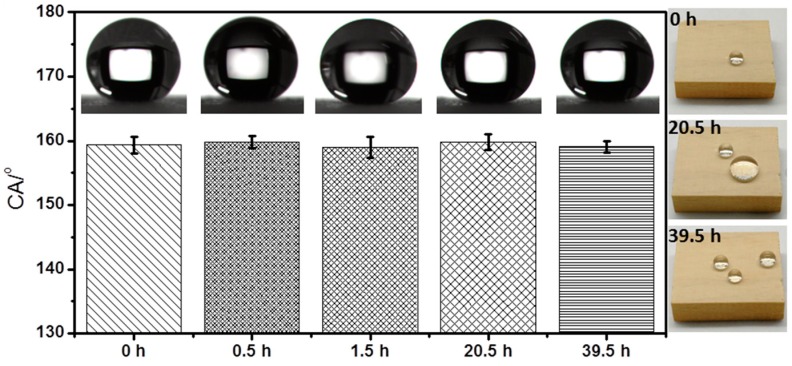
Water CAs and photographs of the coated sanded wood surface after being irradiated with 365 nm UV lamp (11.6 mW∙cm^−2^) for different times.

Wood contains protein, starch, cellulose, etc., which can provide nutrients for microbial growth, so it is easily infected by mildew, especially when the wood surface is wet. Here, the mildew resistance ability of the as-prepared superhydrophobic wood surface was studied and compared with that of the raw wood surface. The results in Figure 7e demonstrate that about 13% of the surface area of the raw wood had been infected by mildew on the 7th day when it was placed in an environment with a humidity of 90% and a temperature of 30 °C, and the percentage of the infected area increased to 100% on the 14th day. By contrast, the coated sanded wood with the superhydrophobic surface was not infected by mildew until the 14th day and only 12% of its surface was infected by mildew on the 28th day under the same environment. The improved mildew resistance could be due to the fact that the superhydrophobic surface repelled water and reduced water adsorption thereby inhibiting the growth of mildew. In addition, this surface can maintain its superhydrophobic ability as shown by only minimal contact angle fluctuations (Figure 8) after a long period of high-intensity UV irradiation which demonstrates the fine UV durability of this superhydrophobic surface. Therefore, it is highly promising to employ this superhydrophobic surface to prolong the service life of wood-based materials.

## 4. Conclusions

In this work, a novel superhydrophobic surface has been fabricated successfully by casting and drying a composite suspension of KH1322 and silica on the modified hierarchical structure of wood. Due to the full use of the natural hierarchical structure of wood, the whole process needs neither complicated equipment nor complex procedures to construct the micro/nano composite structure. Only a very low content of inorganic matter is needed to achieve superhydrophobicity. Furthermore, the fabrication of a superhydrophobic surface can be achieved at room temperature. More importantly, the as-prepared superhydrophobic surface exhibits a satisfactory resistance to abrasion and is able to self-heal at room temperature upon damage. If the healing agents are depleted, the surface can restore its self-healing ability by casting the fluoroalkylsilane/silica composite suspension to replenish the healing agents. When used for wood protection, this superhydrophobic surface greatly improves both mildew inhibition and water resistance of wood, thereby prolonging the service life of wood-based materials. The excellent performance of the as-constructed superhydrophobic surface, the facile and environmentally friendly fabrication process, and the low cost, make this method highly suitable for the protection of various wood-based materials.
